# Multifocal Primary Extranodal Non-Hodgkin Lymphoma: A Report of a Rare Case

**DOI:** 10.7759/cureus.57105

**Published:** 2024-03-28

**Authors:** Vallal Kani, Akhilesh Ravichandran, Sarah Grace Priyadarshini, Nithin Diwagar, Muthuvel Esakki

**Affiliations:** 1 Department of Pathology, Saveetha Medical College and Hospitals, Saveetha Institute of Medical and Technical Sciences, Saveetha University, Chennai, IND

**Keywords:** diffuse large b-cell lymphoma (dlbcl), gastrointestinal lymphoma, lymphoid cells, extranodal lymphomas, non-hodgkin lymphoma (nhl)

## Abstract

Primary extranodal non-Hodgkin lymphoma (NHL) is a rare manifestation of lymphoid malignancies and typically arises in tissues outside the lymph nodes and can involve various organs and anatomical sites presenting unique challenges in diagnosis and treatment. Multifocal primary extranodal NHL, characterized by simultaneous involvement of multiple extranodal sites, presents a diagnostic and therapeutic challenge due to its uncommon presentation and varied clinical manifestations. Despite advances in diagnostic modalities and treatment strategies for NHL, multifocal involvement poses unique clinical dilemmas requiring a multidisciplinary approach for accurate diagnosis and optimal therapeutic intervention. In this case report, we describe a rare case of multifocal primary extranodal NHL in a 29-year-old female, highlighting the complexities associated with its diagnosis and management. Through this case presentation, we aim to underscore the importance of recognizing and addressing the intricacies of multifocal primary extranodal NHL to enhance clinical outcomes and improve patient care. It also highlights the diagnostic complexity and clinical challenges associated with multifocal primary extranodal NHL. A multidisciplinary approach involving haematologists, oncologists, radiologists, and pathologists is crucial for the accurate diagnosis and optimal management of such cases. Additionally, further research is warranted to better understand the underlying pathogenesis and improve treatment strategies for this rare presentation of NHL.

## Introduction

Any form of lymphoma other than Hodgkin lymphoma is included in the diverse category of hematolymphoid neoplasms known as non-Hodgkin lymphoma (NHL) [1]. Although lymphomas found in the small and large intestines are extremely uncommon, primary lymphomas of the extranodal region are most frequently found in the gastrointestinal tract. Around 30-40% of extranodal NHL cases and 10-15% of NHL cases are thought to be caused by uncommon gastrointestinal illness. The stomach is the most common location for gastrointestinal NHL, which makes up 1-4% of gastrointestinal neoplasms. The small intestine and the ileocaecal region are the next most common sites [2,3]. The most prevalent extranodal NHL subtype is diffuse large B-cell lymphoma (DLBCL) of the colon, with the caecum being the most often reported site in the majority of research reports [4]. Here, we report a case of primary extranodal NHL which is a DLBCL subtype occurring simultaneously in the ileum and caecum as involvement of both the small intestine and colon is a very rare condition.

## Case presentation

**Figure 1:**
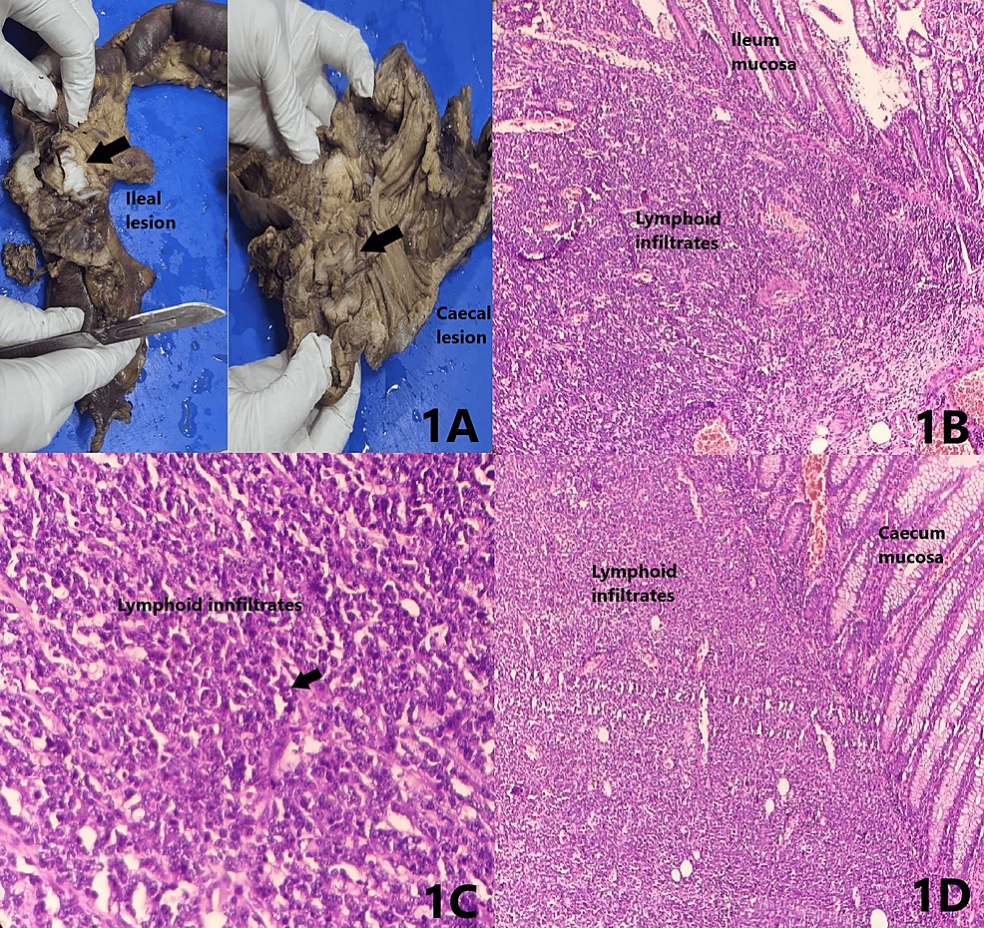
Gross and microscopy Figure 1A: Gross lesion in the bowel (arrows). Figure 1B: Diffuse lymphoid infiltrates in the lamina propria and submucosa of the ileum (10×). Figure 1C: Sheets of large lymphocytes (arrow) in the ileum (40×). Figure 1D: Diffuse lymphoid infiltrates in the lamina propria and submucosa of caecum (10×)

**Figure 2:**
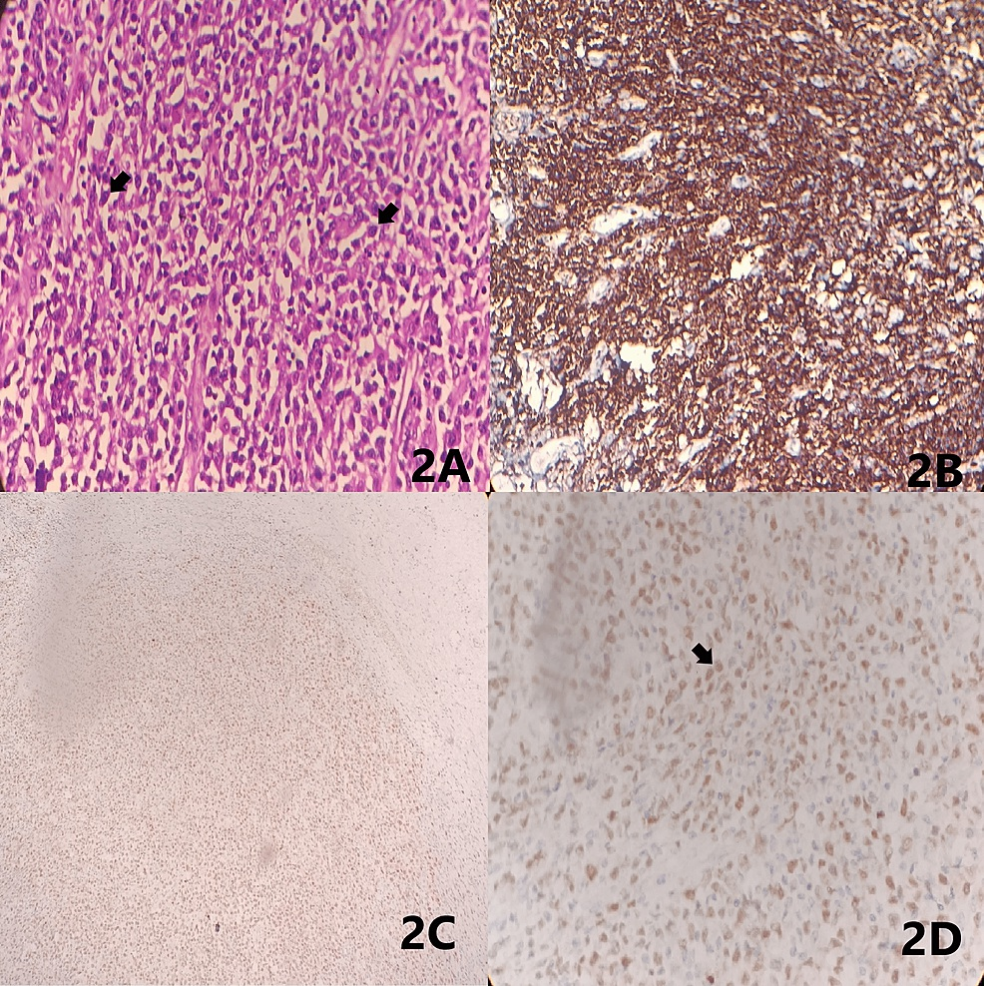
Microscopy with immunohistochemistry Figure 2A: Sheets of large lymphoid cells (arrows) in the caecum (40×). Figure 2B: Immunohistochemistry showing CD45 positivity (20×). Figure 2C: Immunohistochemistry showing multiple MUM1 positivity (4×). Figure 2D: Immunohistochemistry showing multiple MUM1 positivity (arrow) (40×) MUM1: multiple myeloma oncogene 1

A 29-year-old female presented with complaints of recurrent abdominal pain, vomiting, diarrhoea for three days, and weight loss for three weeks following which examination revealed a palpable mass in the abdomen. Vitals and baseline investigations were found to be normal. The clinical impression was given as subacute intestinal obstruction after confirming radiologically. Right hemicolectomy with repair surgery was done, and the specimen was received for histopathological examination. Macroscopically, the specimen was irregular showing adhesions at the ileocaecal junction. The cut surface revealed two lesions, one in the ileum and the other in the caecum. The ileum showed a stricturing lesion (tumour) measuring 3.5 cm which was 21.5 cm from the ileocaecal junction. The caecum showed a polypoidal lesion which was 2 cm from the ileocaecal junction (Figure 1A). Microscopically, multiple sections from the ileal and colonic mucosa showed a neoplasm composed of sheets of atypical, medium- to large-sized lymphoid cells with moderately pleomorphic, vesicular nuclei, visible nucleoli, and scant eosinophilic cytoplasm. Occasional mitotic figures were seen (6-7 per 10 high-power fields). The surrounding stroma shows small lymphocytes, plasma cells, and occasional mast cells (Figure 1B, 1C, 1D and Figure 2A). On immunohistochemistry, the tumour cells were diffusely membrane positive for CD45 (Figure 2B), and multiple myeloma oncogene 1 (MUM1) shows diffuse (>90%) strong nuclear staining of the tumour cells (Figure 2C, 2D). Based on the above findings, a final diagnosis of DLBCL non-germinal center subtype was made.

## Discussion

**Figure 3:**
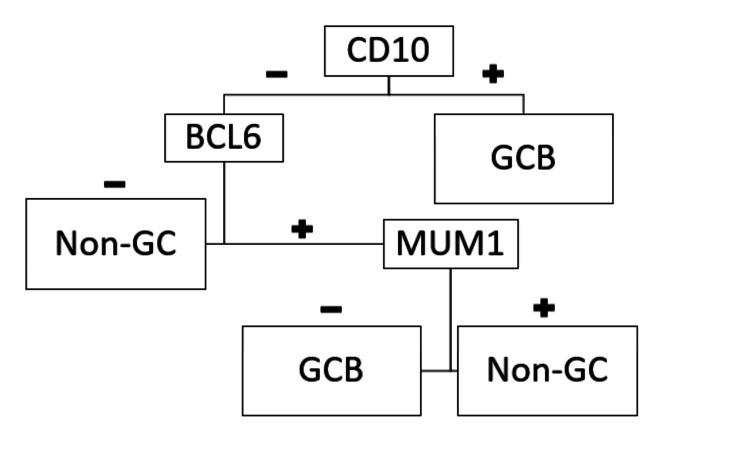
Hans algorithm CD10: cluster of differentiation 10; BCL6: B-cell lymphoma 6; MUM1: multiple myeloma oncogene 1; GCB: germinal center B cell; Non-GC: non-germinal center

NHL is a malignant tumour that falls under the category of lymphoproliferative disorders. It develops from B cells, T cells, or natural killer cells at different stages of maturation. More than 30 subtypes of NHL exist, with follicular lymphoma (FL) and DLBCL being the most prevalent forms [3]. Because this ailment is uncommon, it is vital to understand how it presents, progresses, and is managed. It has a male predominance and is usually diagnosed in the fifth to seventh decade [3,5]. DLBCL and mantle cell lymphoma are the two histological forms of colorectal lymphoma that occur most frequently [5]. As far as we are aware, our case is one of the very few instances of gastrointestinal lymphoma that have been documented in the literature including several gastrointestinal tract locations (ileum and caecum). Battah et al. have documented a unique case of DLBCL that concurrently affects the ascending colon and the duodenum [6]. Also, an unusual case of DLBCL occurring primarily in the ovary and small intestine has been reported [7]. DLBCL of the colon and rectum is associated with no specific risk factors. However, several predisposing factors have been identified like advanced age, autoimmune disorders, immunodeficiency states (human immunodeficiency virus, organ transplant), inflammatory bowel disease, celiac disease, *Helicobacter pylori*, etc. Proto-oncogene activation and chromosomal abnormalities in band 3q27 are part of the pathophysiology of colorectal lymphomas. In 35% of DLBCL patients, B-cell lymphoma 6 (BCL6) gene rearrangement is the primary factor. The BCL6 gene plays an important role in germinal center formation, modifying lymphocyte function and lymphocyte differentiation [3,4]. Presenting features may vary including abdominal pain, weight loss, night sweats, fever, lower gastrointestinal bleeding, diarrhoea, colonic obstruction, and, in rare cases, colonic perforation. A proper diagnosis is made by biopsy of the lesion during endoscopy or after histopathological examination of the specimen received after bowel resection or post-colectomy [4]. Several radiological tests, including positron emission tomography-computed tomography (PET-CT), magnetic resonance imaging (MRI), and computed tomography (CT) are important for assessing the disease's stage and extent. When a patient presents with neurological manifestations, brain imaging such as CT, MRI, or lumbar puncture may be necessary. A bone marrow biopsy may also be necessary, depending on the extent and stage of the disease, to rule out the possibility of lymphoma involving the marrow and to distinguish between primary and secondary cases of lymphomas [6]. The Dawson criteria can be helpful in diagnosing primary gastrointestinal lymphoma which includes no peripheral lymph node enlargement at the time of presentation, absence of lymphadenopathy of the mediastinal region, normal leucocyte count, predominant lesions in the bowel with only adjacent lymph node involvement at the time of surgery, and absence of lymphomatous involvement of the liver and spleen [8]. DLBCL is clinically and biologically heterogeneous with wide variation in the survival rate of the patients. Clinical characteristics like the International Prognostic Index (IPI) and gene expression profiling influence the prognosis of DLBCL. Numerous researches on the varied prognostic measures of DLBCL have been published. Using complementary deoxyribonucleic acid (cDNA) microarray technology, DLBCL has been divided into prognostically relevant subgroups: germinal center B cells (GCB) and activated B cells (ABC). Since performing microarray analysis on every patient with DLBCL is both unfeasible and costly, several immunohistochemistry (IHC) techniques have been developed to convert the solid data from molecular research into routine clinical data for use. IHC-based algorithms created by Hans and his associates were first commonly utilized to categorize DLBCL into the germinal center and non-germinal center subtypes. The cluster of differentiation 10 (CD10), BCL6, and MUM1 protein immunohistochemical expressions serve as the foundation for the algorithm (Figure 3).

Hans algorithm IHC has the benefit of requiring only three readily assessable antibodies, making it more commonly accepted than other algorithms that were later created to subtype DLBCL based on the cell of origin. Research revealed that the Hans algorithm clearly distinguished between the GCB and ABC DLBCL groups in terms of survival and had a strong correlation with the matching gene expression profile results [9,10]. Gastrointestinal lymphoma was staged using a modified version of the Ann Arbor staging system, which was widely utilized as shown in Table 1 [11]. Treatment for gastrointestinal lymphoma depends on an accurate staging and diagnosis.

**Table 1:** Ann Arbor staging system of lymphoma

Stage of the disease	Features
Stage I	A single lymph node or an extranodal location
Stage II	Two or more lymphatic regions on the same side of the diaphragm or a single extranodal organ with lymph node involvement on the same side of the diaphragm
Stage IIA	Involvement of regional lymph nodes
Stage IIB	Involvement of distant lymph nodes
Stage III	Lymph node involvement on both sides of the diaphragm
Stage IV	Disseminated disease with involvement of other extranodal sites (like the bone marrow, liver, or abdominal wall)

The treatment option requires a multidisciplinary approach consisting of chemotherapy, radiotherapy, surgery, or their combinations. The drug regimen consists of cyclophosphamide, vincristine, doxorubicin, and prednisone (CHOP) which is a combination of anthracyclines used in the mainstay chemotherapy. An anti-CD20 monoclonal antibody called rituximab is currently approved for use in the therapy. It has been discovered that the combination of CHOP regimen and rituximab is more beneficial than monotherapy, resulting in full remission and cure in the majority of patients. Additionally, it has enhanced the course of treatment and long-term survival percentage for DLBCL patients [3,6].

## Conclusions

The case presented herein exemplifies the diagnostic challenges and therapeutic complexities associated with multifocal primary extranodal NHL. Through a comprehensive diagnostic workup involving imaging studies, endoscopic evaluations, histopathological examination, and molecular analyses, a definitive diagnosis of DLBCL was established in our patient. The management of multifocal primary extranodal NHL requires a multidisciplinary team comprising haematologists, oncologists, radiologists, and pathologists to optimize treatment strategies and enhance patient outcomes. Given the rarity of this presentation, further research is warranted to elucidate the underlying pathogenesis and identify novel therapeutic targets for improved patient care. By sharing our experience, we hope to contribute to the existing body of literature and facilitate better understanding and management of this rare manifestation of lymphoid malignancy.
